# Effects of Metabolic Syndrome with or without Obesity on Outcomes after Coronary Artery Bypass Graft. A Cohort and 5-Year Study

**DOI:** 10.1371/journal.pone.0117671

**Published:** 2015-02-13

**Authors:** Hushan Ao, Fei Xu, Xianqiang Wang, Xinran Tang, Zhe Zheng, Shengshou Hu

**Affiliations:** Department of Anesthesiology, State Key Laboratory of Translational Cardiovascular Medicine, Fuwai Hospital and Cardiovascular Institute, the Chinese Academy of Medical Sciences, Beijing, China; INRCA, ITALY

## Abstract

**Background:**

Metabolic syndrome (MetS) and obesity are risk factors for cardiovascular disease, however, it remains unclear about effects of MetS with or without obesity on perioperative and long-term morbidity and mortality after coronary artery bypass graft (CABG).

**Methods:**

An observational cohort study was performed on 4,916 consecutive patients receiving isolated primary CABG in Fuwai hospital. Of all patients, 1238 patients met the inclusion criteria and were divided into three groups: control, MetS with obesity and MetS without obesity (n = 868, 76 and 294 respectively). The patient’s 5-year survival and major adverse cerebral and cardiovascular events (MACCE) were studied.

**Results:**

Among all three groups, there were no significant differences in in-hospital postoperative complications, epinephrine use, stroke, ICU stay, ventilation time, atrial fibrillation, renal failure, coma, myocardial infarction, repeated revascularization, and long-term stroke. The patients in MetS without obesity group were not associated with increased perioperative or long-term morbidities and mortality. In contrast, the patients in MetS with obesity group were associated with significant increased perioperative complications including MACCE (30.26% vs. 20.75%, 16.7%, p = 0.0074) and mortality (11.84% vs. 3.74%, 3.11%, p = 0.0007) respectively. Patients in MetS with obesity group was associated with significantly increased long-term of MACCE (adjusted OR:2.040; 95%CI:1.196–3.481; P＜0.05) and 5-years of mortality (adjusted HR:4.659; 95%CI:1.966–11.042; P＜0.05).

**Conclusions:**

Patients with metabolic syndrome and obesity are associated with significant increased perioperative and long-term complications and mortality, while metabolic syndrome without obesity do not worsen outcomes after CABG.

## Introduction

Diabetes and obesity are increasingly more prevalent among patients undergoing coronary artery bypass grafting (CABG) [1,2]. Coronary heart disease (CAD) is the leading cause of death among the population with these risk factors. The metabolic syndrome (MetS) is a cluster of metabolic perturbations largely resulting from an excess accumulation of abdominal fat, and it is characterized by insulin resistance, hypertriglyceridemia, low high-density lipoprotein cholesterol, and the presence of small dense low-density lipoprotein particles [3]. MetS has been shown to result in a dramatic increase of type 2 diabetes and cardiovascular disease (CVD) [4]. Several previous reports suggested that the pro-inflammatory and pro-thrombotic features of MetS could adversely affect the surgical outcomes in patients undergoing CABG [5]. Also, the metabolic perturbations of the viscerally obese patient are accompanied by the presence of oxidative stress which is the pathogenic mechanism linking to MetS [6].

Several previous studies have reported about the effects of MetS on patients with CAD, with special connection to diabetes, obesity, and hypertension [7–9]. There is a consensus that MetS is much more than just the sum of its components or a singular factor, it exposes patients to a higher risk of complications. In addition, the higher levels of inflammatory markers found in patients with MetS lead to the low-grade inflammatory state in these patients, which exposes them to subclinical atherosclerosis and development of cardiovascular complications. Several papers have shown that MetS is an independent risk factor for both early and late mortality and morbidity in patients with CAD undergoing percutaneous coronary intervention [7–10]. There were reports on effects of MetS on the early postoperative outcome in patients undergoing CABG [11–13]. Ivanovic et al [14] reported that patients with four or five MetS criteria had a marginally increased risk of the MACCE than the subjects with three MetS criteria in the 3-year follow-up. Nonetheless, it remains unclear concerning which component of MetS is a more important predictor for mortality and morbidity after CABG, especially lacking of long-term of clinical study on outcomes in those patients. It is well known that obesity, especially central obesity is one of key features of MetS. The present study aimed to determine the impact of MetS with or without obesity on perioperative and long-term outcomes in patients undergoing CABG.

## Materials and Methods

### Study population

**Fig 1:**
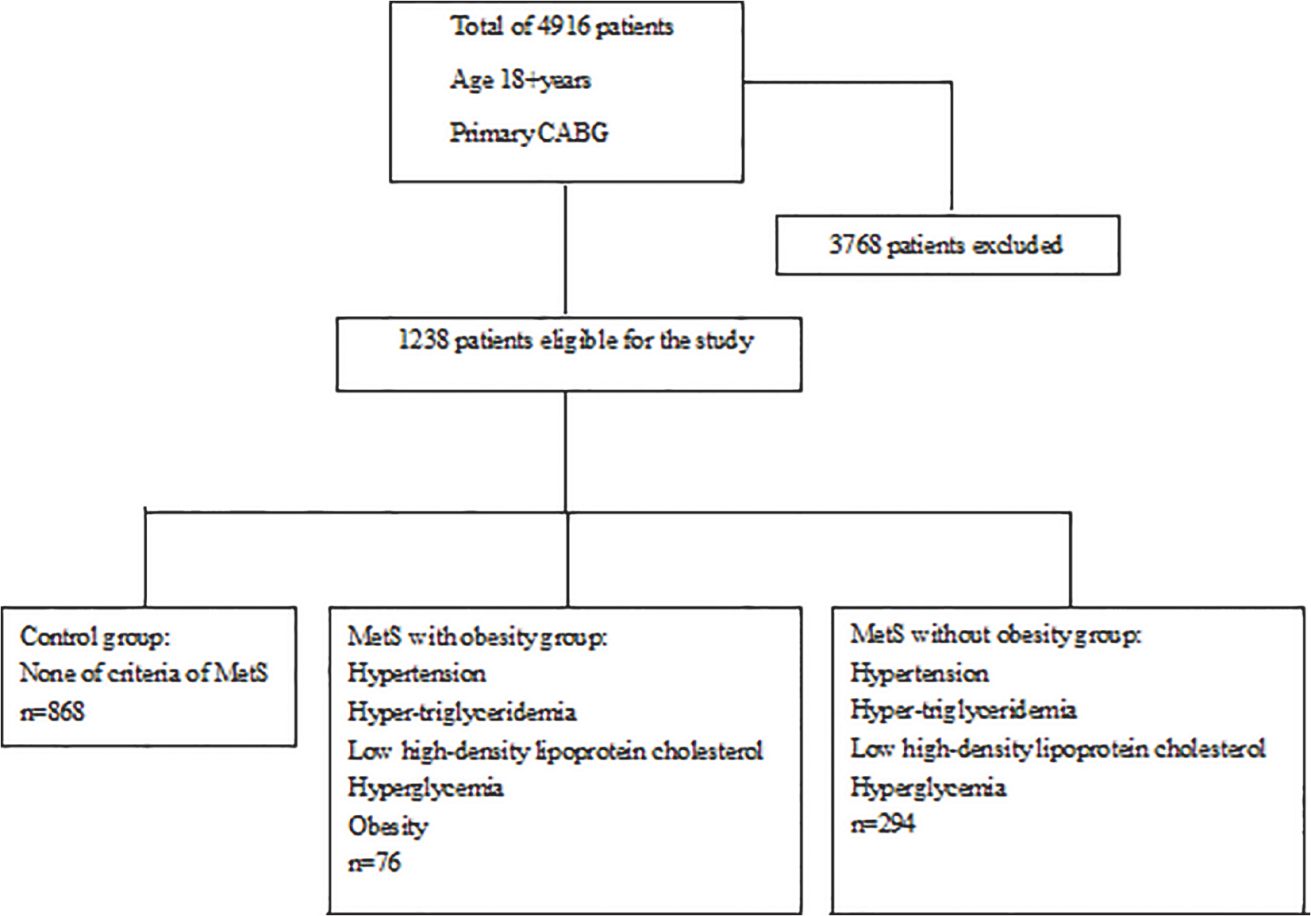
Study Population Recruitment Summary.

This study was a cohort, retrospective study involving 4,916 consecutive patients receiving isolated primary CABG at Fuwai Hospital in Beijing, China, dated from January 1, 1999 to December 30, 2005. The study was in compliance with the Declaration of Helsinki, approved by Fuwai Hospital Institutional Review Board and the institutional review board waived the need for written informed consent from the participants in compliance with the waiver criteria. All patient records and information were anonymized and de-identified prior to analysis. All the extracted patients were in a Chinese population. Of all patients, 1,238 met the inclusion criteria and were divided into three groups: control group (n = 868), MetS with obesity group (n = 76) and MetS without obesity group (n = 294). Control group means patients combined with none of criteria of MetS. In this study, MetS is defined as the patient with hypertension, hyper-triglyceridemia, low high-density lipoprotein cholesterol, hyperglycemia and with or without obesity. MetS with obesity group means patients combined with hypertension, hyper-triglyceridemia, low high-density lipoprotein cholesterol, hyperglycemia and obesity. MetS without obesity group means patients combined with hypertension, hyper-triglyceridemia, low high-density lipoprotein cholesterol and hyperglycemia. (Fig. 1). The 5-year survival and MACCE were studied.

### Operative techniques

The anesthetic and surgical techniques were standardized for all patients. Patients were operated through a mid-sternotomy. After surgery, patients were transferred to the intensive care unit. They were extubated as soon as they met the following criteria: normothermia, awake, hemodynamic stability, and no significant bleeding.

### Identification of patients with the metabolic syndrome

The clinical diagnosis of the MetS was made by using the modified Adult Treatment Panel III of the National Cholesterol Education Program that requires at least three of the following [15,16]: obesity: body mass index (BMI) greater than 28kg/m^2^, hyper-triglyceridemia: elevated triglycerides(>150mg/dL in women or drug treatment), low high-density lipoprotein cholesterol: reduced high-density lipoprotein cholesterol (<40 mg/dL in men, <50 mg/dL in women or drug treatment), hypertension: elevated arterial blood pressure(>130mmHg systolic, >85mmHg diastolic or drug treatment), and hyperglycemia: elevated fasting glucose (FPG>100 mg/dL or drug treatment).The original criterion to classify obesity in the Adult Treatment Panel III was waist circumference >120cm in men,>88cm in women, but this was not measured in this cohort. Therefore we used BMI to substitute for waist circumference. Recent studies showed that most patients identified as having MetS based on the BMI would also have been diagnosed as obese according to waist circumference [17,18].

### Outcome events definition

Renal failure was defined as a need for dialysis to treat prolonged oliguria or anuria [19]; stroke as central neurological deficit persisting more than 72h; coma as being unresponsive for more than 24h; encephalopathy as reversible neurological deficit (recovery within 72h of onset); low cardiac output syndrome (LCOS) as cardiac index lower than 2.01/min/m^2^ and left ventricular assist(LVAD),intra-aortic balloon pump (IABP) and inotropic support after surgery. Outcomes were recorded from follow-up included death and MACCE.MACCE was defined as permanent or transient stroke, coma, perioperative myocardial infarction (MI),heart block, and cardiac arrest [20–22].

### Statistical Analysis

Continuous variables were described as mean ± standard deviation, and compared by ANOVA analysis. Categorical variables were described as frequency and percentages, and compared by the chi-square test or Fisher’s exact test. Cumulative incidence of mortalities of each group at 5 years was estimated by Kaplan-Meier method and compared by log-rank test. Univariate Cox regression and multiple Cox regression models were used to analysis follow-up mortalities. Univariate logistic regression and multiple logistic regression models were used to analysis MACCE. Covariates included in the multiple regression models were baseline variables that showed significant difference among 3 groups (including age, sex, smoking, aortic cross-clamp time, cerebrovascular events, peripheral artery disease, thrombolytic therapy, myocardial infarction and left main disease). All statistical tests were two-sided, and the significant level is 0.05 and performed with the SAS 9.13 software (SAS Institute, Cary, NC, USA).

## Results

### Baseline and intraoperative characteristics

Of 4916 eligible patients in the database, 1238 patients met the inclusion criteria and were divided into three groups as showed in Fig. 1. Demographic and clinical data of the patients are presented in Table 1. No significant differences were found among three groups in history of renal failure, myocardial infarction, left main disease, heart failure, atrial fibrillation, preoperative creatinine, intraoperative LVEF, intravenous nitrate, cardiopulmonary bypass time, and aortic cross-clamp time. However, the patients of MetS without obesity tended to be older (61.50±7.72) and more likely to be females (27.21%), more with history of cerebrovascular events (10.88%vs.7.89%, 2.53%.P<0.0001), peripheral artery disease (13.61%vs.5.26%, 7.26%.P = 0.0021), diseased coronary artery (2.84±0.40). Patients with MetS and obesity were more likely to have smoking (61.84%), unstable angina pectoris (22.37%), using intravenous nitrate (0.13±0.35). Control group were more likely to have thrombolytic therapy (12.90%) and history of myocardial infarction (51.96%). (Table 1)

**Table 1:** Baseline Characteristics of the patients.

Variables	MetS without Obesity	MetS with Obesity	No MetS	P Value
	N = 294	N = 76	N = 868	
Age (yrs.)	61.50±7.72	58.08±7.72	58.95±9.48	<0.0001
BMI(kg/m^2^)	24.70±2.17	32.96±24.77	24.19±2.26	<0.0001
Female	80(27.21%)	15(19.74)	98(11.29)	<0.0001
Smoking	130(44.22%)	47(61.84%)	489(56.34%)	0.0005
Family History	31(10.54%)	5(6.58%)	54(6.22%)	0.0463
Hypertension	294(100%)	76(100%)	0	<0.0001
Hyperlipidemia	294(100%)	76(100%)	0	<0.0001
Elevated fasting glucose	294(100%)	76(100%)	0	<0.0001
History of Renal Failure	0	0	2(0.23%)	0.6525
Creatinine(μmol/L)	98.89±102.08	133.75±196.34	110.38±123.25	0.4174
Cerebrovascular Events	32(10.88%)	6(7.89%)	22(2.53%)	<0.0001
Peripheral Artery Disease	40(13.61%)	4(5.26%)	63(7.26%)	0.0021
Thrombolytic Therapy	19(6.46%)	4(5.26%)	112(12.90%)	0.0024
Angina Pectoris				
Unstable Angina Pectoris	50(17.01%)	17(22.37%)	59(6.80%)	<0.001
Stable Angina Pectoris	18(6.12%)	4(5.26%)	27(3.11%)	0.061
Myocardial Infarction	129(43.88%)	38(50.00%)	451(51.96%)	0.0568
Diseased Coronary Artery	2.84±0.40	2.72±0.48	2.71±0.57	0.0008
Left Main Disease	84(28.57%)	20(26.32%)	270(31.11%)	0.5348
Heart Failure	4(1.36%)	2(2.63%)	18(2.07%)	0.6730
Atrial Fibrillation	5(1.70%)	3(3.95%)	15(1.73%)	0.3791
LVEF	59.90±9.62	58.41±8.36	59.15±10.04	0.3847
Intravenous Nitrate	0.07±0.26	0.13±0.35	0.05±0.22	0.0062
Cardiopulmonary bypass time(min)	65.93±62.86	63.88±64.48	58.48±76.78	0.2939
Aortic cross-clamp time(min)	42.29±40.43	41.37±42.58	36.39±40.29	0.0751

BMI: body mass index; LVEF: left ventricular ejection fraction.

### Perioperative outcomes

As showed in Table 2, among three groups there were no differences in in-hospital postoperative complications, epinephrine use, stroke, ICU stay, ventilation time, atrial fibrillation, renal failure, coma, myocardial infarction, repeated revascularization and stroke. Patients with MetS were more likely to have death (11.84% vs. 3.74%, 3.11% respectively, p = 0.0007) and MACCE (30.26% vs.20.75%, 16.7%respectively, p = 0.0074) than other groups.

**Table 2:** Post-operative Characteristics.

Variables	MetS without Obesity	MetS with Obesity	No MetS	P Value
	N = 294	N = 76	N = 868	
Postoperative Complications	3(1.02%)	0	7(0.81%)	0.6754
Epinephrine	35(11.90%)	14(18.42%)	93(10.71%)	0.1250
Stroke	3(1.02%)	1(1.32%)	4(0.46%)	0.4412
Duration of ICU Stay(h)	61.11±82.09	48.26±38.29	61.06±63.92	0.2770
Ventilation Time(h)	19.53±34.45	16.22±7.12	15.86±24.17	0.1166
Atrial Fibrillation	31(10.54%)	4(5.26%)	62(7.14%)	0.1188
Renal Failure	3(1.02%)	1(1.32%)	1(0.12%)	0.0462
Coma	1(0.34%)	0	7(0.81%)	0.5298
Mortality	11(3.74%)	9(11.84%)	27(3.11%)	0.0007
Myocardial Infarction	5(1.70%)	2(2.63%)	8(0.92%)	0.2898
Repeated Revascularization	8(2.72%)	3(3.95%)	33(3.80%)	0.6754
Stroke	41(13.95%)	11(14.47%)	86(9.91%)	0.1044
MACCE	61(20.75%)	23(30.26%)	145(16.7%)	0.0074

ICU: intensive care unit; MACCE: major adverse cardiac and cerebrovascular events.

### The long-term mortality

**Fig 2:**
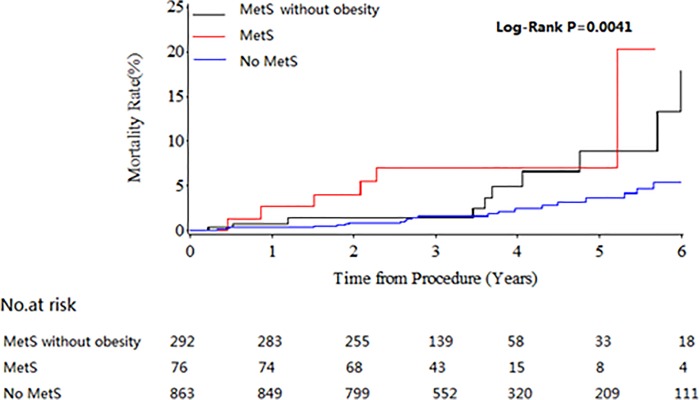
Kaplan-Meier for Mortality Rate Following-up Five Years.

The median follow up duration was 59.3 months in this cohort population. Compared to control group, patients in MetS with obesity group were associated with significantly increased long-term mortality (HR 3.621; 95%CI 1.576–8.319; P = 0.0024) while the mortality was found no significant difference in MetS without obesity group (Table 3). The Kaplan-Meier curves in Fig. 2 illustrate the trend of long-term mortality among three groups. And MetS with obesity showed significant higher mortality than other groups (Log-rank test P = 0.0077). Multivariable Cox regression model including all covariates was used to adjust the confounding factors and analyze the association among MetS, obesity and mortality. As showed in Table 4, patients in MetS with obesity group are associated with increased long-term follow-up mortality (adjusted HR:4.659;95%CI:1.966–11.042;P<0.05), in addition, old age is also a risk factor for the long-term mortality (adjusted HR:1.051;95%CI:1.014–1.091;P<0.05).There were no significant differences found in MetS without obesity group.

**Table 3:** MetS and Long-term Mortality.

Variables	Un-adjusted			Adjusted		
	HR	95%CI of HR	P Value	HR	95%CI of HR	P Value
MetS vs. No MetS	3.621	1.576–8.319	0.0024	4.659	1.966–11.042	0.0005
MetS without obesity vs. No MetS	1.834	0.901–3.735	0.0945	1.376	0.637–2.969	0.4167

HR: hazard ratio; CI: confidence interval.

**Table 4:** Multivariable-Adjusted Hazard Ratio of Long-term Mortality.

Variables	P Value	HR	HR95% CI
MetS	0.005	4.659	1.966–11.042
MetS without Obesity	0.4167	1.376	0.637–2.969
Age	0.0074	1.051	1.014–1.091
Sex	0.1906	0.539	0.214–1.360
Smoking	0.1268	1.702	0.860–3.367
Cerebrovascular Events	0.0989	2.307	0.855–6.227
Peripheral Artery Disease	0.1019	2.181	0.857–5.551
Thrombolytic Therapy	0.3856	1.457	0.622–3.413
Myocardial Infarction	0.9067	0.962	0.502–1.842
Left Main Disease	0.8781	0.952	0.506–1.791

### The Long-term follow-up MACCE

Compared with control group, patients in MetS with obesity group were associated with significantly increased long-term MACCE (OR 2.164; 95%CI 1,285–3.643; P = 0.0155). In contrast, patients in MetS without obesity group did not show difference in the mortality. Multivariable logistic regression model was used to adjust the confounding factors to analysis long-term MACCE. As in Table 5, MetS with obesity is associated with increased the rate of MACCE (adjusted OR:2.040;95%CI:1.196–3.481;P<0.05). In addition, old age (adjusted OR: 1.026; 95%CI: 1.008–1.045; P<0.05) and cerebrovascular events (adjusted OR: 1.990; 95%CI: 1.116–3.547; P<0.05) are also risk factors for the rate of MACCE. (Table 6)

**Table 5:** MetS and MACCE in the 5-year follow-up.

Variables	Un-adjusted			Adjusted		
	OR	95%CI of OR	P Value	OR	95%CI of OR	P Value
MetS vs. No MetS	2.164	1.285–3.643	0.0155	2.040	1.196–3.481	0.0133
MetS without obesity vs. No MetS	1.305	0.935–1.822	0.5419	1.093	0.766–1.559	0.1908

OR: odds ratio; CI: confidence interval.

**Table 6:** Multivariable-Adjusted Odd Ratio of Long-term MACCE.

Variables	P-Value	OR	OR95%CI
MetS	0.0133	2.040	1.196–3.481
MetS without Obesity	0.1908	1.093	0.766–1.559
Age	0.0039	1.026	1.008–1.045
Sex	0.2862	0.791	0.513–1.218
Smoking	0.0824	1.335	0.964–1.850
Cerebrovascular Events	0.0197	1.990	1.116–3.547
Peripheral Artery Disease	0.1926	1.385	0.848–2.262
Thrombolytic Therapy	0.9054	0.970	0.584–1.610
Myocardial Infarction	0.7832	1.044	0.768–1.420
Left Main Disease	0.7977	0.959	0.697–1.320

## Discussion

This study demonstrated that MetS with obesity is an important predictor for higher morbidity and mortality in patients with CABG up to 5-years postoperatively. In contrast, MetS without obesity was not found to be independent predictors of operative and long-term mortality as well as MACCE in this study, indicating that obesity is an important and critical component of MetS in determining patients’ outcomes after CABG. The results also suggest that the metabolic abnormalities associated with MetS with obesity have a much greater negative impact on long-term outcome after CABG than MetS without obesity does.

As the epidemic of overweight and sedentary lifestyle grows worldwide, the metabolic syndrome is becoming increasingly common, resulting in an enormous economic burden to the society [23–25]. Knowledge of the impact of the metabolic syndrome on cardiovascular and overall mortality in the general population is crucial for developing public health policy and clinical guidelines for its prevention and treatment. The findings of the present study indicate that many Chinese adults have MetS and obesity. CVD is already the leading cause of death in China, and without a national emphasis on the prevention and control of obesity and the metabolic syndrome, the burden of this problem in China is likely to increase in the near future. Obesity is generally associated with an increased operative morbidity [26]. A meta-analysis of BMI effect on all-cause mortality and incidence of CVD showed that a reduction of overweight (BMI to<24.0kg/m^2^) might reduce the incidence of stroke by 15% in men and 22% in women in China [27]. However, Echahidi et al [11] did not found that obesity is independent predictors of operative mortality in their study on patients with CABG. We recently also found that obesity were not significantly associated with 5-years mortality and MACCE in ethnic Chinese patients undergoing CABG [28]. Very few studies have previously attempted to delineate the role of MetS and/or obesity on the long-term MACCE and mortality. Therefore, this study aimed to investigate the impact of MetS with or without obesity on outcomes in the Chinese patients undergoing CABG

A previous study reported that the prevalence of CAD is relatively low in obesity patients without MetS, but it is markedly increased among obesity patients with MetS [29] suggesting that the metabolic perturbations associated with MetS are key factors in the development of atherosclerosis and cardiovascular events. In this study [29], MetS without obesity had no impact on MACCE and mortality even other four components of MetS including elevating triglycerides, reduced high-density lipoprotein cholesterol and elevated arterial blood pressure existed. As Echahidi et al [11] found, diabetes or obesity per se was not found to be an independent predictor of operative mortality for CABG, however, patients with diabetes and MetS had a markedly increased risk of postoperative mortality, whereas those without MetS were not, indicating that diabetes is an important risk component of MetS for patients undergoing CABG. Nonetheless, it remains unclear concerning the role of obesity and its potential long-term prognostic effect in patients undergoing CABG.

This study provides further evidence that obesity is an essential component of MetS in contributing to the adverse long-term outcomes in the Chinese patients undergoing CABG. The finding has major clinical implications. For instance, MetS is a potentially preventable and modifiable condition that often goes undiagnosed and untreated. MetS is highly prevalent (40% to 50%) in the population of CABG patients and may contribute worsening outcomes. The identification of MetS could thus be useful to classify high-risk patients for CABG, in addition, the integration of MetS, especially associated with obesity into the operative risk algorithms might improve risk stratification for cardiac patients. MetS and obesity is prevalent and serious risk factors for long-term mortality after CABG surgery as showed in this and other studies. Thus, optimal medical therapy and appropriate lifestyle of the behavior therapy could lead to a controlled form of MetS and obesity, potentially improving long-term outcomes after CABG.

In addition, the sub-analysis of the present study showed that old age and history of cerebrovascular events were risk factors for MACCE after CABG. Further studies to address those risk factors, including diabetes, obesity and other components in MetS are important in order to improve outcomes of patients with MetS undergoing CABG. As a previous study showed, cerebral complications could significantly increase operative mortality and severely limit postoperative recovery and quality of life [30].

**Limitations.** This is a retrospective study in single center in the ethnic Chinese patients. As a retrospective study, the potential problems of a non-randomized study may remain despite of multivariate adjustment was used in this study to reduce overt biases. Also, MetS was assessed using BMI because waist circumferences were not available in this cohort of patients. Although international guidelines suggest the use of waist circumference to classify obesity, several studies have demonstrated that there is no significant difference between the two classification methods, i.e., BMI or waist circumference.

## Supporting Information

S1 Dataset All relevant data within the manuscript. (XLSX)
